# Development of sparse Bayesian multinomial generalized linear model for multi-class prediction

**DOI:** 10.1186/1471-2105-15-S10-P14

**Published:** 2014-09-29

**Authors:** Behrouz Madahian, Lih Y Deng, Ramin Homayouni

**Affiliations:** 1 Department of Mathematical Sciences, University of Memphis, Memphis, TN, 38152, USA; 2 Bioinformatics Program, University of Memphis, Memphis, TN, 38152, USA; 3 Department of Biology, University of Memphis, Memphis, TN 38152, USA

## Background

Gene expression profiling has been used for many years to classify samples and to gain insights into the molecular mechanisms of phenotypes and diseases. A major challenge in expression analysis is caused by the large number of variables assessed compared to relatively small sample sizes. In addition, identification of markers that accurately predict multiple classes of samples, such as those involved in the progression of cancer or other diseases, remains difficult.

## Materials and methods

In this study, we developed a multinomial Probit Bayesian model which utilized the double exponential prior to induce shrinkage and reduce the number of covariates in the model [1,2]. A fully Bayesian hierarchical model was developed in order to facilitate Gibbs sampling which takes into account the progressive nature of the response variable. Gibbs sampling was performed in R for 100k iterations and the first 20k were discarded as burn-in. The method was applied to a published dataset on prostate cancer progression downloaded from Gene Expression Omnibus at NCBI (GSE6099) [3]. The data set contained 99 prostate cancer cell types in four different progressive stages. The dataset was randomly divided into training (N=50) and test (N=49) groups such that each group contained an equal number of each cell type. Before applying our model, for each gene we performed ordinal logistic regression. Genes were ranked based on the p-value of association. Using a cutoff value of 0.05 after Benjamini and Hochberg FDR correction resulted in a final set of 398 genes.

## Results

**Figure 1:**
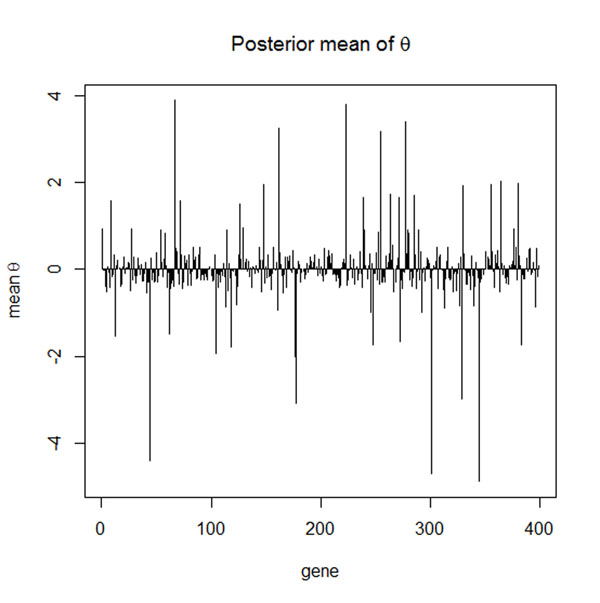
Posterior mean of θs associated with gene1 to gene 398.

Figure 1 shows the posterior mean of parameters associated with each gene. Using the top ten genes obtained from our model, we were able to achieve 86% classification accuracy in the training group and 82% accuracy in the test group. To test the robustness of the model, we switched the training and test groups and evaluated the classification accuracy. We obtained 88% classification accuracy on the new training group and 86% accuracy on the new test group. The classification accuracy by tumor type is shown in Table 1. Taken together, these results suggest that the Bayesian Multinomial Probit model applied to cancer progression data allows for reasonable subclass prediction.

**Table 1:** Classification accuracy of prostate cancer subtypes in the train and test groups.

Sample type	Train group	Test group	Train group Switched	Test group switched
**Benign**	100	94	100	100
**PIN**	29	33	50	71
**PCA**	86	86	100	100
**MET**	100	100	70	50

## Conclusion

Our future plan is to perform resampling on the selection of training and test groups in order to obtain more robust results and to compare the performance of the model to other popular classifiers such as Support Vector Machine and Random Forest.
